# The Influence of Selected Factors on Changes in Locomotion Activity during Estrus in Dairy Cows

**DOI:** 10.3390/ani14101421

**Published:** 2024-05-09

**Authors:** Mária Mičiaková, Peter Strapák, Eva Strapáková

**Affiliations:** 1 Institute of Animal Husbandry, Slovak University of Agriculture in Nitra, Trieda Andreja Hlinku 2, 949 76 Nitra, Slovakia; peter.strapak@uniag.sk; 2 Institute of Nutrition and Genomics, Slovak University of Agriculture in Nitra, Trieda Andreja Hlinku 2, 949 76 Nitra, Slovakia; eva.strapakova@uniag.sk

**Keywords:** estrus, locomotion activity, dairy cows, selected factors

## Abstract

**Simple Summary:**

Ensuring regular reproduction is crucial for successful dairy farming. However, increased milk production demands and efforts to enhance productivity have adversely affected fertility indicators. Fertility disorders are currently significant reasons for culling dairy cows from herds. Accurate estrus detection, based on a complex of physiological signs and behaviors preceding ovulation, is fundamental for achieving desired reproductive outcomes. Successful fertilization hinges on optimal insemination and ovulation timing. The Heatime RuminAct system stands out as a leading technology for estrus detection in cows. It leverages biological principles of cow behavior during estrus, notably, increased locomotion activity. Our study confirms this, showing a substantial (*p* < 0.001) 33% rise in the locomotion activity of Holstein dairy cows during estrus compared to the reference period 3 days prior, an average increase of 278 u.24 h^−1^. Post-estrus, locomotion activity dropped significantly on the first day, gradually stabilizing to levels akin to pre-estrus. Through statistical analysis, we explored how parity, lactation stage, milk yield, and individuality affect locomotion activity during estrus and the reference period. Our findings underscored parity’s notable impact (F = 13.41, *p* < 0.001) on altering dairy cows’ locomotion patterns during estrus.

**Abstract:**

The objective of this study was the evaluation of the locomotion activity of heifers and Holstein dairy cows during estrus. We have analyzed the locomotion activity using the Heatime RuminAct device on 180 cows (32 heifers and 148 dairy cows) and we evaluated a total of 633 estrus cycles during the reference period of 3 days before estrus, 3 days after estrus, and on the day ofestrus occurrence. The datawere analyzed using the DataFlowTM II program. The locomotion of cows was expressed in the units of locomotion activity in 24 h (u.24 h^−1^). During the reference period of 3 days before estrus, the cows showed locomotion activity of 558 u.24 h^−1^, with an increase in locomotion activity on the day of estrus of 836 u.24 h^−1^, and, during the reference period of 3 days after estrus, the level of locomotion activity decreased to 537 836 u.24 h^−1^, which is a similar level of locomotion activity to the reference period before estrus. Through the statistical analysis, we evaluated the impact of parity, lactation stage, milk yield, and individuality on changes in locomotion activity during estrus and throughout the reference period, and we found a significant effect of parity (F = 13.41, *p* < 0.001) on changes in the locomotion activity of dairy cows during estrus. Based on these results, this research offers fresh perspectives on assessing specific factors affecting the locomotion activity of dairy cows during estrus through the practical application of electronic systems for estrus detection on dairy farms.

## 1. Introduction

Dairy farms encounter numerous challenges and transformations. As herd sizes increase and the number of farmers or employees per cow decreases, the need arises for innovative strategies to uphold or enhance reproductive management [1,2]. Detecting estrus in a herd is a crucial aspect of herd reproduction management [3]. Breeders and researchers focus on accurate estrus identification based on external symptoms because missing estrus can lead to significant economic losses for breeders [4,5]. Previous experiments have demonstrated that the estrous period in dairy cows is characterized by increased locomotion activity, serving as a reliable tool for accurate estrus detection [6,7,8]. This increased activity is also a prerequisite for achieving the desired insemination results. The primary reason for heightened activity during estrus may be the gradual increase in estrogen concentration (17β-estradiol) in the cows’ blood before the first estrus (proestrus). Lopez et. al. [9] and Lyimo et al. [10] highlight the significant relationship between estradiol concentration in dairy cows’ blood and estrus length (r = 0.57), as well as estradiol concentration and changes in dairy cows’ behavior during the estrus period (r = 0.7).

To enhance estrus detection effectiveness, various electronic technologies have been developed [11], along with additional equipment measuring changes in cows’ behavior during estrus [7]. Currently, there is widespread use of different electronic systems evaluating locomotion activity and other systems targeting the assessment of cattle estrus symptoms [11,12]. According to the authors, commercially available electronic equipment for estrus detection aims to record changes in locomotion activity measured by a pedometer placed on the limb [13,14] and on the dam’s neck [15,16]. According to Rorie et al. [11], Firk et al. [7], and Roelofs et al. [17], electronic devices for detecting estrus in dairy cows have the widest application. Reith and Hoy [18] recommended giving the highest priority to detection based on sensor-supported activity monitoring, with this being the most successful tool for automated estrus detection.

One of the most modern technologies for estrus detection nowadays is the Heatime RuminAct system. The system is considered a highly effective tool for detecting estrus in females. The system is based on the biological principles of cows’ behavior during estrus, characterized by increased locomotion activity and a simultaneously reduced rumination level, which is influenced by a physiological decrease in feed intake during estrus. Based on the evaluation of these two indicators, the system can provide basic information to the breeder in real-time about nutrition, animal behavior, comfort, and overall health status of the cows 24 h per day, 7 days per week, in real-time. Therefore, the Heatime RuminAct system represents a modern and unique tool for breeders, allowing for up to 95% accuracy in estrus detection, including identifying silent estrus in dairy cows [19].

In this study, we aimed to evaluate the impact of estrus on changes in the locomotion activity of dairy cows. In addition, based on statistical analysis, we assessed the influence of selected factors parity, lactation stage, milk yield, and the animal (individuality) on changes in the locomotion activity of dairy cows during the reference period (before estrus and after estrus) compared to the values of these indicators during estrus.

## 2. Materials and Methods

### 2.1. Animals and Housing

Baseline measurements were conducted on two herds, one with 350 and the other with 120 Holstein dairy cows. For Herd 1, dairy cows are housed in free-stall barns with cubicles, utilizing manure solid bedding. Stable floors are made of concrete with rubber mats. Animals are provided Total Mixed Ration (TMR) ad libitum throughout lactation. The ration includes corn and alfalfa silage as roughage components and a nuclear mixture to supplement the energy and protein requirements of the animals. Core feeds/kernel feeds are allocated based on performance achieved and the classification of animals into production groups. Additionally, dairy cows are given corn silage meal, protected fat, and mineral feed additives to supplement their ration. Milking is carried out thrice daily in the Boumatic double-12 milking parlor. Dairy cows wear Heatime RuminAct collars around their necks from calving until pregnancy is confirmed.

Herd 2 also utilizes free-stall housing for dairy cows with straw-bedded cubicles. Heifers are kept freely on deep bedding, which is changed every 20 days. The stall floors are made of concrete. Animals are provided Total Mixed Rations (TMR) ad libitum throughout lactation. The animals receive a partial total mixed ration with the following components: grass silage, maize silage, core mixture, corn flakes, hay, molasses, and minerals. Diets are offered twice daily at 0700 ± 1 h and 1700 ± 1 h for ad libitum intake. Cows are milked twice per day (0800 and 1900 h) in a double-5 milking parlor and are kept away from feed during milking. The average milk yield of Herd 1 is 10,140 kg, and for Herd 2 is 9430 kg of milk per lactation. Dairy cows wear Heatime RuminAct collars around their necks from the age of 13 months until they are culled from the herd.

Dairy cows were inseminated for the first time at 63 and 84 days after calving, respectively. We recorded the length of the service period (days open) on average from 113 days (Herd 1) to 121 days (Herd 2). The pregnancy rate for Herd 1 was 42.29% and for Herd 2 on average 48.76%. Herd management identified estrus through activity measurement and visual observation. Locomotion activity was recorded using Heatime RuminAct monitoring (SCR Engineers Ltd., Netanya, Israel). The cows calved year-round and were artificially inseminated by technicians from the AI association (Herd 1) and by the farm manager (Herd 2). Pregnancy diagnosis was conducted by ultrasonography between 25 and 30 days after AI and by rectal palpation at 60 ± 5 days after AI.

### 2.2. Data Collection

We assessed the locomotion activity of 180 cows (32 heifers and 148 dairy cows) of the Holstein breed. In total, 633 estrus cycles were available for study. Data were collected between July 2019 and November 2022. The day when the cow was artificially inseminated was defined as estrus (d0), where the period from midnight to midnight was considered ‘a day’. The day when we recorded the so-called estrus peak, which the technology producer defines as a change in locomotion activity compared to the preceding seven-time interval, was considered estrus. The system calculates the “heat index” (estrus peak index) for estrus detection, identifying a cow as in estrus when the index value ranges from 35 to 100. 

### 2.3. Measurement of Locomotion Activity

For the measurement of locomotion activity and rumination time, all cows were equipped with the Heatime RuminAct monitoring system (SCR Engineers Ltd., Netanya, Israel) attached to the neck collar of the cow. An acceleration sensor continuously recorded individual cow locomotion activity and calculated the activity index in ‘activity units’ (u.24 h^−1^). Basic activity data were analyzed in a microprocessor by complex algorithms that separated the cow’s day-to-day activity from activities associated with estrus behavior. The data stored in 2 h intervals were read by an antenna and automatically transferred in real-time to the herd management software program DataFlow II. The 2 h values were arithmetically averaged to one value per day for further analysis (u.24 h^−1^). variables.

We conducted evaluations every day in the reference period of 3 days before estrus, on the day of estrus, and every day in the reference period of 3 days after the estrus completion. The estrus day was considered to be the day of insemination.

In assessing the influence of selected factors on changes in the locomotion activity of dairy cows, we analyzed 633 estrus cycles by parity (0-heifers, n = 78; 1-primiparous cows, n = 237; multiparous cows on the second and next lactation, n = 318). According to the stage of lactation, we divided the dairy cows into 1st, within 80 days of calving (n = 236), 2nd, from 81 to 150 days of calving (n = 180), and 3rd, over 151 days of calving (n = 139). The lactation stage was defined as the number of days from calving to the onset of estrus. The range of days for the lactation stage factor was specified in the herd management software. Based on daily milk yield, we divided the group of dairy cows into two groups: low milk yield dairy cows, < 34.29 kg of milk per day (n = 277), and high milk yield dairy cows, ≥ 34.29 kg of milk per day (n = 356). Milk yield was calculated as an average daily milk yield 10 days before estrus to eliminate any influence of approaching estrus on milk yield changes. We conducted evaluations every day in the reference period of 3 days before estrus, on the day of estrus, and every day in the reference period of 3 days after the estrus completion by parity and milk yield.

### 2.4. Statistical Analysis

For the preparation and statistical processing of the data, we utilized the statistical program SAS 9.3, Enterprise Guide 5.1 (SAS Institute Inc., Cary, NC, United States, 2011). Regarding the locomotion activity indices under evaluation, we computed basic statistical characteristics considering parity, lactation stage, and average daily milk yield, determined 10 days before the onset of estrus.

Using the *t*-test, we examined differences in locomotion activity between the designated groups of dairy cows based on the order of lactation, stage of lactation, and average milk yield on each day of the reference period and the day of estrus.

Changes in locomotion activity during the reference period and on the day of estrus were calculated using analysis of variance (ANOVA), and the significance of the means was tested through Scheffe’s test.

The impact of factors such as herd, parity, lactation stage, milk yield, and individuality on changes in the locomotion activity of dairy cows at estrus compared to the reference period (3 days before estrus and 3 days after estrus) was analyzed using linear models as described by model equations.

Y_ijklm_ = μ + H_i_ + P_j_ + SL_k_ + M_l_ + I_m_ + e_ijklm_

where:

Y_ijklm_ represents the change in locomotion activity over time during estrus compared to the reference period.

μ is the intercept, the overall mean.

H_i_ is the fixed effect of the herd, with n = 2 levels (Herd 1, Herd 2).

P_j_ is the effect of parity, with n = 3 levels (0-heifers, 1-primiparous cows, 2-multiparous cows).

SL_k_ is the effect of the lactation stage, with n = 4 levels (0-heifers, 1-within 80 days after calving, 2—from 81 to 150 days after calving, and 3-over 151 days after calving).

M_l_ is the effect of milk yield, with n = 2 levels (1-less than 34.29 kg, 2-greater and equal to 34.29 kg).

I_m_ is the effect of individuals, with n = 180 females.

e_ijklm_ represents the residual error, which accounts for random effects or unobserved factors.

The Post Hoc Tests (Duncan’s test) were used to compare changes in locomotion activity across different levels of assessed factors.

## 3. Results

### 3.1. The Influence of Estrus on Changes in the Locomotion Activity of Dairy cows

**Figure 1:**
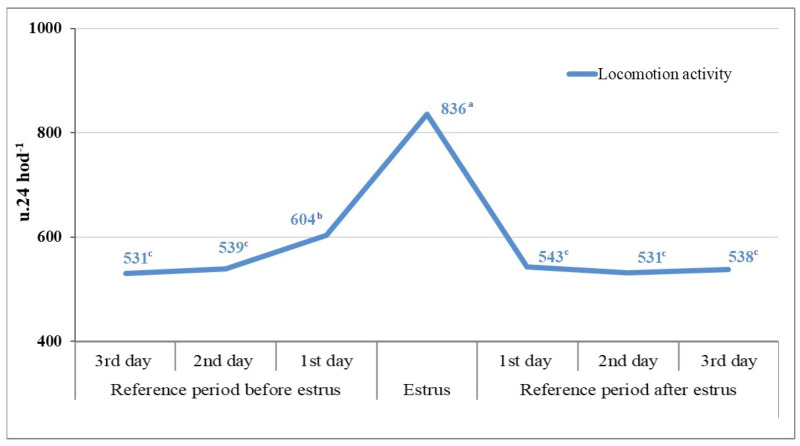
Changes in the locomotion activity of dairy cows in the reference period and during estrus (n = 633). ^a–c^-the values marked with different letters are statistically significant (*p* < 0.05).

When evaluating the impact of estrus on changes in the locomotion activity of the dairy cows, we found an average activity of 558 u.24 h^−1^ in the reference period of 3 days before estrus. The onset of full estrus led to a statistically significant increase (*p* < 0.001) in the locomotion activity of the dairy cows up to 836 u.24 h^−1^, representing an increase of +278 u.24 h^−1^ (+33%) compared to the reference period before estrus and an increase of +27.7% compared to the values of locomotion activity 1 day before estrus (Figure 1). This more significant increase in locomotion activity of the females one day before estrus was likely influenced by the onset of estrus itself, with a gradual increase in the intensity of external estrus signs, which were conditioned by the rising concentration of estrogen in the blood. After the end of estrus, we observed an immediate and significant decrease in the locomotion activity of the dairy cows to +543 u.24 h^−1^ on day 1 post-estrus (Figure 1).

### 3.2. The Influence of Parity on Changes in the Locomotion Activity of Cows during Estrus

**Figure 2:**
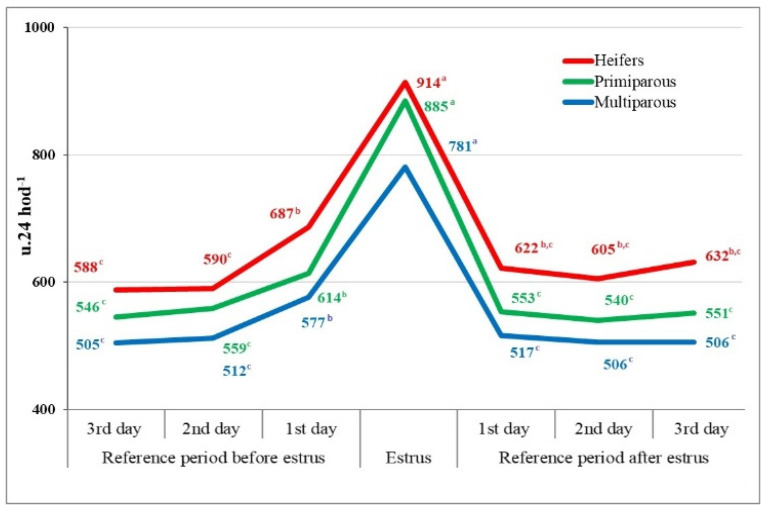
Changes in the locomotion activity of dairy cows in the reference period and during estrus according to parity (n = 633). ^a–c^-the values labeled with distinct letters exhibit statistical significance (*p* < 0.05).

A more significant increase in locomotion activity in the heifer group to 687 u.24 h^−1^ was recorded 1 day before estrus, representing an increase compared to the previous period of +98 u.24 h^−1^. Similarly, in the group of assessed heifers, as well as in the group of primiparous and older cows, 1 day before estrus, a more pronounced increase in the locomotion activity for 24 h was observed at 614 and 577 u.24 h^−1^, respectively (Figure 2).

In connection with the onset of estrus, marked by the occurrence of peak activity in the estrous cycle, heifers experienced an increase in locomotion activity for 24 h to 914 u.24 h^−1^ (by +227 u.24 h^−1^, or +32%), in the group of assessed primiparous cows to 855 u.24 h^−1^ (by +274 u.24 h^−1^, or +30%), and in older cows to 781 u.24 h^−1^, which was the lowest increase in locomotion activity compared to the value of this indicator 1 day before estrus (by +204 u.24 h^−1^, or +20%) (Figure 2). The highest differences in locomotion activity in the group of assessed heifers could be explained by the fact that heifers are not burdened with milk yield, have lower weight, and, physiologically, their organism is more active compared to the organism of older cows. The lowest differences in the increase in locomotion activity in the category of multiparous cows during estrus may be related to higher milk yield, more intense blood circulation, and, thus, faster elimination of estrogens from the blood, which conditions a quicker decrease in estrogen concentration in the blood of older cows, and, consequently, a reduction in the intensity of external signs of estrus.

From the results of testing the differences in the locomotion activity of heifers and primiparous cows over 24 h in the reference period and during estrus, concerning age and parity, statistically significant differences (*p* < 0.001, resp. *p* < 0.05) were calculated on the 3rd, 2nd, and 1st days of the reference period before estrus between heifers and primiparous cows, heifers and multiparous cows, as well as between primiparous cows and multiparous cows. Differences in locomotion activity between the heifer and primiparous cow group were not statistically significant on any day of the reference period before estrus (Table 1). These trends were also confirmed on the day of estrus, where statistically significant differences (*p* < 0.001) were calculated between heifers and multiparous cows, as well as between the evaluated primiparous cows and multiparous cows. In the reference period after the completion of estrus, based on the analysis conducted, statistically significant differences in locomotion activity (*p* < 0.001) were found between heifers, primiparous cows, and multiparous cows on all 3 days of the reference period (Table 1).

Based on the results of the study and the performed statistical analysis, we can conclude that individual categories of dairy cows exhibited varying intensities of estrus behavior, as assessed based on locomotion activity.

### 3.3. The Influence of Lactation Stage on Changes in the Locomotion Activity of Dairy Cows

As part of the analysis of selected factors, we also assessed the locomotion activity of Holstein dairy cows based on the lactation stage the number of days post-calving.

**Figure 3:**
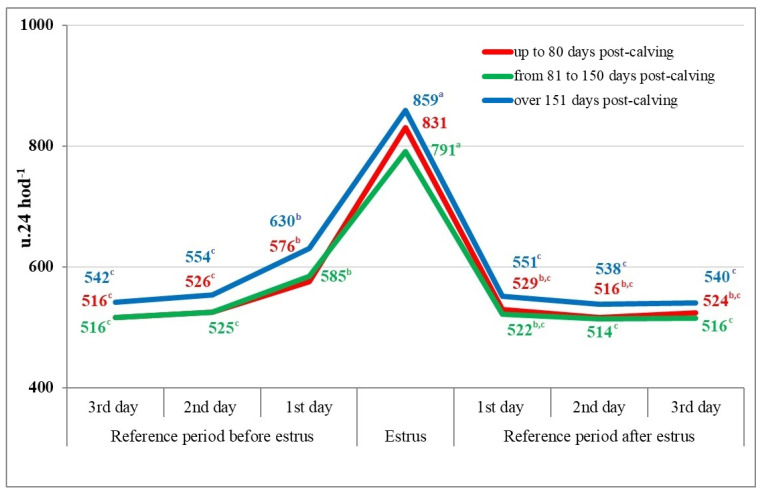
Changes in the locomotion activity of dairy cows in the reference period and during estrus according to the lactation stage (n = 556). ^a–c^-the values labeled with distinct letters exhibit statistical significance (*p* < 0.05).

A more significant increase in the locomotion activity of dairy cows during the reference period was observed one day before estrus, which was confirmed in all evaluated groups of cows according to the lactation stage by +50 and +60, respectively, and +76 units per 24 h in the group of cows over 151 days post-calving (Figure 3).

In connection with the onset of estrus, a significant and relatively consistent increase in the locomotion activity of cows was calculated between the day before estrus and the day of estrus, ranging from +206 units per 24 h in the 2nd group of cows (80–150 days post-calving) to +255 units per 24 h in the 1st group of cows (up to 80 days post-calving) (Figure 3).

During the reference period after the end of estrus, a reduction in locomotion activity over 24 h was observed on the first day after estrus in all groups of cows, divided according to the lactation stage, reaching levels of 529 and 522, respectively, and 551 units per 24 h, with subsequent mild decreases in this indicator on the following days (2nd and 3rd day of the reference period) after the end of estrus (Figure 3).

In the statistical analysis of testing differences in 24 h locomotion activity according to the defined lactation stage, statistically significant differences (*p* < 0.05, resp. *p* < 0.01) were calculated during the reference period before estrus between the 1st group of cows (up to 80 days post-calving) and the group of cows over 151 days post-calving, as well as the 2nd group of cows (81 to 150 days post-calving) and the 3rd group of cows (over 151 days post-calving) (Table 2).

During estrus and on each day of the reference period after the end of estrus, statistically significant differences (*p* < 0.01) were confirmed only between the 2nd and 3rd group of tested cows according to the lactation stage. Among the other evaluated groups of cows, according to the lactation stage, there were no statistically significant differences during the reference period before estrus, after the end of estrus, as well as during estrus (Table 2).

### 3.4. The Influence of Milk Yield on Changes in the Locomotion Activity of Dairy Cows during Estrus

In terms of evaluating the impact of milk yield on the level of locomotion activity, we divided all cows into two main groups cows with lower milk yield and high-yielding cows. In the reference period before estrus, the assessed groups of cows -cows with lower daily milk yields (up to 34.29 kg) and high-yielding cows (over and equal to 34.29 kg of milk) exhibited a comparable level of locomotion activity, reaching levels of from 524 to 529 movement units (u.24 h^−1^) on the 3rd and 2nd day before estrus, respectively, or from 536 to 548 u.24 h^−1^. One day before estrus, which can be considered the beginning of estrus due to the effect of estrogens, there was a slight increase in the locomotion activity of individual groups of cows to 589 and 616 u.24 h^−1^, respectively.

**Figure 4:**
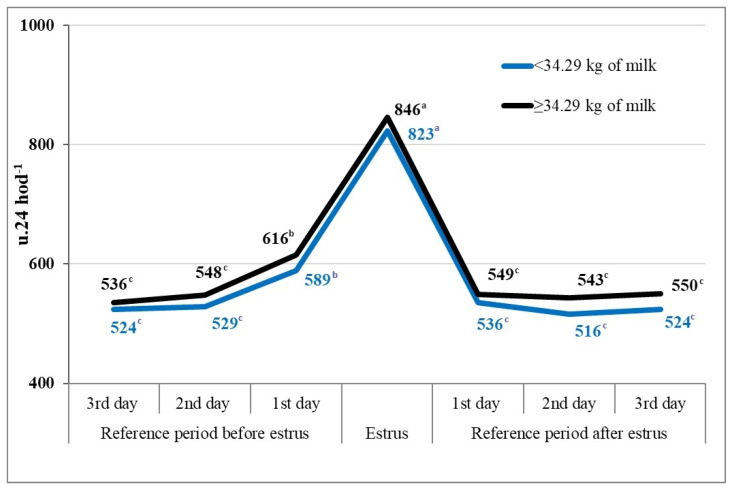
Changes in locomotion activity of cows in the reference period and during estrus according to milk yield (n = 556). ^a–c^-the values labeled with distinct letters exhibit statistical significance (*p* < 0.05).

As anticipated, during estrus, there was a notable surge in locomotion activity observed in cows with lower milk yields, reaching 823 u.24 h^−1^ (+34%), and in high-yielding cows, reaching 846 u.24 h^−1^ (+33%). The discrepancy between the assessed groups of cows amounted to 23 u.24 h^−1^ (3%). According to the study findings, greater locomotion activity in high-yielding cows (exceeding or equaling 34.29 kg of milk) was reaffirmed both in the reference period before estrus and during estrus. This increase was paralleled in both groups of cows during estrus compared to the locomotion activity recorded one day before estrus (by +234 and +230 u.24 h^−1^, respectively) (Figure 4). Likewise, following the conclusion of estrus, during the reference period of three days post-estrus, we generally observed higher locomotion activity in the high-yielding cows (ranging from 543 to 550 u.24 h^−1^) compared to in the cows with lower milk yields (ranging from 516 to 536 u.24 h^−1^) (Figure 4).

Based on the statistical analysis of differences in locomotion activity over 24 h among individual groups of cows according to the achieved average milk yield (below 34.29 kg and above and equal to 34.29 kg) in the reference period before estrus, during estrus, and after the end of estrus, statistically significant differences (*p* < 0.05) were calculated for the 2nd and 1st days of the reference period before estrus, and significance at the *p* < 0.01 level was confirmed on the 2nd and 3rd days after the end of estrus. On the other days of the reference period and on the day of estrus itself, statistically significant differences in locomotion activity between cows with lower and higher milk yields were not confirmed (Table 3).

### 3.5. Statistical Analysis of the Influence of Selected Factors on Changes in Locomotion Activity of Dairy Cows in the Reference Period and during Estrus

Within the statistical analysis, we investigated the impact of selected factors herd, parity, lactation stage, and milk yield on changes in the locomotion activity of cows in the reference period (before estrus and after the end of estrus) compared to the values of this indicator during estrus (Table 4).

Based on the linear model, we calculated the significant influence of parity (F = 13.41, *p* <0.001) on changes in the locomotion activity of cows during estrus compared to the reference period.

Based on the results of Post Hoc Tests, we observed significant differences in locomotion activity during estrus primiparous and multiparous cows (Table 5).

## 4. Discussion

Lopez et al. [9] state that the most likely reason for the increase in locomotion activity during estrus may be the gradual elevation of estrogen (17β estradiol) concentration in the blood of dairy cows during the preparation period for estrus (proestrus). The authors found the maximum estrogen concentration one day before estrus, 16 h before estrus, and 9 h after the end of estrus [20]. Our study’s results, in connection with the increased activity of cows, also corroborate the conclusions of Arney et al. [21] and Zebari et al. [22], who noted a steady and consistent rise in the activity levels of dairy cows three days preceding estrus, followed by a more pronounced surge in activity leading up to the onset of estrus, and Reith et al. [23], who documented heightened activity as early as two days before estrus.

Regarding the impact of estrus on changes in locomotion activity, our study did not confirm a 2- to 4-fold increase in the activity of cows during estrus compared to the reference period before estrus, as reported by Kiddy [24] and Arney et al. [21]. Similarly, Reith et al. [25] confirmed, in connection with the onset of estrus, an increase in the locomotion activity of Holstein and Fleckvieh dairy cows by an average of +38%. Comparable results of increased locomotion activity in Holstein dairy cows during estrus compared to the reference period before estrus were also found based on our study results (+33%) [13]. Silper et al. [19] reported significantly higher differences in locomotion activity when comparing the pre-estrus period with the values of this indicator during estrus, expressed by the level of activity in 2 h time intervals (u.2 h^−1^) at a level of 77.3 u.2 h^−1^, respectively, and Madureira et al. [26], who found the average activity of dairy cows during estrus at the level of 72.8 u.2 h^−1^. Firk et al. [12] present fundamentally divergent outcomes from the experiments conducted, and Kerbrat and Disenhaus [27], on the other hand, did not report a more notable elevation in cows’ locomotion activity during estrus.

The assessment of the decrease in the locomotion activity of dairy cows after the end of estrus also corroborates the conclusions by Reith et al. [23], who reported a noticeable decrease in this indicator already on the 1st day after the end of estrus, which, according to the authors, is closely related to the decrease in the concentration of estradiol in the blood of dairy cows.

Berka et al. [28] and Roelofs et al. [13] registered the most significant rise in locomotion activity during estrus in the category of heifers compared to older cows, a finding confirmed by the results of our study. In the evaluated heifers, there was an increase in locomotion activity of +274 units per 24 h in connection with the onset of estrus, whereas, in older cows, this increase amounted to only +204 units per 24 h. Similar conclusions were also confirmed by Madureira et al. [26], who calculated the average activity level of heifers during estrus as 77.3 units of the index value for 2 h (u.2 h^−1^), and, for older cows, as 72.8 index values (when converted to 24 h, this represents a difference of 54 units per 24 h). A more pronounced increase in the locomotion activity of heifers during estrus was also found based on experiments conducted by Walker et al. [29], Lopez et al. [9], Roelofs et al. [13], Yániz et al. [14], and Reith et al. [25], who point to a lower intensity of locomotion activity in heifers compared to older cows, which is also in line with the results of our study. In contrast, van Vliet and van Eerdenburg [30] confirmed higher locomotion activity in older cows in the 2nd and subsequent lactations compared to the group of evaluated heifers. The issue of testing the influence of cow age, parity, milk yield level, and lactation stage on the increase in locomotion activity during estrus was also addressed by Arney et al. [21]. Based on conducted experiments, the authors found that the activity of cows increases in parity, which is fundamentally contrary to the results of our study, where, in our experiment, heifers exhibited an increase in locomotion activity of +274 units per 24 h during estrus, while older cows showed an increase of only +204 units per 24 h. This may be related to the fact that the heifer’s body is not burdened by a high level of milk production.

The issue of testing the influence of the lactation stage on changes in the locomotion activity of cows during estrus was also addressed by Arney et al. [21], who found that the locomotion activity of cows decreases during the advancing lactation stage post-calving. Based on the results of our study, we can conclude that the highest level of locomotion activity during the reference period before estrus (average 575 units per 24 h), during estrus (859 units per 24 h), and after the end of estrus (average 543 units per 24 h) was exhibited by cows in the lactation stage over 151 days post-calving, which did not confirm the conclusions of the authors Arney et al. [21].

López-Gatius et al. [31] and Yániz et al. [14] point to a negative relationship between milk yield and the intensity of locomotion activity during estrus. They verified that milk yields notably impact both the duration of estrus and the concentration of estradiol in cows’ blood. In contrast, based on the results of our study, we can state that cows with higher milk yields (greater and equal to 34.29 kg) exhibited non-significantly higher levels of locomotion activity in both the reference period and during estrus (by +23 u.24 h^−1^).

The impact of milk yield on the level of locomotion activity in cows during the period around estrus was also analyzed by Reith et al. [23], who observed statistically significant differences in the locomotion activity of all evaluated groups of cows between the reference period and the day of insemination (estrus). Additionally, the authors found an increase in the locomotion activity of cows with lower (up to 40 kg) and higher milk yields (above 40 kg), confirming the results of our study.

Arney et al. [21], based on the results of their experiments, stated that the achieved milk yield did not significantly influence the level of locomotion activity in cows during estrus. This aligns with the assessment of changes in cows’ locomotion activity during estrus considering our study’s achieved milk yield. On the other hand, Lopez et al. [9] recorded lower locomotion activity in high-yielding cows (above 39.5 kg of milk), and these cows also exhibited a lower concentration of estradiol in the blood on the day of estrus, resulting in less pronounced estrus manifestations compared to cows with lower milk yields.

According to López-Gatius [31], each increase in milk yield by 1 kg reduces locomotion activity during estrus by −1.6%. Likewise, Reith et al. [25] noted that the rise in activity among high-yielding cows (producing over 40 kg of milk per day) was comparatively less prominent than in cows with lower milk yields. These trends were not confirmed in our analysis, as we observed a relatively consistent increase in the locomotion activity of cows with lower milk yields (up to 34.29 kg) and high-yielding cows (above and equal to 34.29 kg of milk) between the last day of the reference period and estrus by +234 and +230 units of locomotion activity (u.24 h^−1^), representing an increase of +34% and +33%, respectively.

After conducting the study, we can conclude that, in recent years, the physiology of female reproduction has undergone significant changes, accompanied by a general decline in fertility indicators, which may be influenced by physiological adaptation to high milk yield. Critical areas of new research include the control of metabolic influences of lactation on the fertility of dairy cows, monitoring estrous cycles, as well as mechanisms related to reproductive disorders and early embryonic mortality.

An essential factor for the desired indicators of dairy cow management and reproduction is effective and accurate estrus detection. One of the characteristic behaviors of dairy cows in estrus is an increase in locomotion activity, as confirmed by the results of our study. These findings provide new and original insights into the evaluation of selected influences on the level of locomotion activity in dairy cows during estrus using the Heatime RuminAct system under practical conditions on dairy farms.

## 5. Conclusions

Based on the results of our study, a statistically significant increase in the locomotion activity of females during estrus (by 33%) compared to the reference period before and after estrus was confirmed, indicating the significant influence of estrogen concentration on changes in the behavior of dairy cows during estrus. When evaluating the impact of selected factors parity, lactation stage, milk yield, and individuality based on the performed statistical analysis, we found a significant effect of parity on changes in the locomotion activity of dairy cows during estrus. For the other factors lactation stage, milk yield, and individuality of animal our hypothesis was not confirmed.

## Figures and Tables

**Table 1:** Testing differences in the locomotion activity of cows in the reference period and during estrus according to parity.

Indicator		Number of Lactation	Difference of Averages	*t*-Test	*p*
Reference period before estrus	the 3rd day	0–1	42.2	2.64	<0.05
		0–2	82.6	5.57	<0.001
		1–2	40.4	4.05	<0.001
	the 2nd day	0–1	30.6	1.98	NS
		0–2	77.7	5.4	<0.001
		1–2	47.0	4.84	<0.001
	the 1st day	0–1	73.1	2.99	<0.01
		0–2	110.2	4.67	<0.001
		1–2	37.2	2.67	<0.01
Estrus		0–1	28,9	0.96	NS
		0–2	132,7	4.6	<0.001
		1–2	103,8	5.39	<0.001
Reference period after estrus	the 1st day	0–1	69.2	4.25	<0.001
		0–2	105.7	6.98	<0.001
		1–2	36.5	3.46	<0.001
	the 2nd day	0–1	65.0	4.36	<0.001
		0–2	99.4	7.06	<0.001
		1–2	34.4	3.53	<0.001
	the 3rd day	0–1	80.1	5.58	<0.001
		0–2	125.7	9.63	<0.001
		1–2	45.6	4.43	<0.001

0-heifers; 1-primiparous cows; 2-multiparous cows; *p*-significance.

**Table 2:** Testing differences in the locomotion activity of cows in the reference period and during estrus according to the lactation stage.

Indicator		Stage of Lactation	Difference of Averages	*t*-Test	*p*
Reference period before estrus	the 3rd day	1–2	0.03	0.0	NS
		1–3	−25.88	−2.06	<0.05
		2–3	−25.91	−1.97	<0.05
	the 2nd day	1–2	0.91	0.08	NS
		1–3	−27.91	−2.28	<0.05
		2–3	−28.82	−2.22	<0.05
	the 1st day	1–2	−8.78	−0.54	NS
		1–3	−54.23	−3.23	<0.01
		2–3	−45.45	−2.47	<0.05
Estrus		1–2	39.52	1.73	NS
		1–3	−37.75	−1.17	NS
		2–3	−67.36	−2.73	<0.01
Reference period after estrus	the 1st day	1–2	7.47	0.64	NS
		1–3	−21.98	−1.64	NS
		2–3	−29.45	−2.18	<0.05
	the 2nd day	1–2	2.40	0.22	NS
		1–3	−21.96	−1.8	NS
		2–3	−24.36	−1.99	<0.05
	the 3rd day	1–2	8.11	0.69	NS
		1–3	−16.38	−1.29	NS
		2–3	−24.49	−1.77	NS

1-cows up to 80 days post-calving; 2-cows from 81 to 150 days post-calving; 3-cows over 151 days post-calving, *p*-significance.

**Table 3:** Testing differences in locomotion activity of cows in the reference period and during estrus according to milk yield.

Indicator		Difference of Averages	*t*-Test	*p*
Reference period before estrus	the 3rd day	11.9	1.27	NS
	the 2nd day	19.3	2.11	<0.05
	the 1st day	27.2	2.02	<0.05
Estrus		23.5	1.27	NS
Reference period after estrus	the 1st day	13.4	1.34	NS
	the 2nd day	27.0	2.92	<0.01
	the 3rd day	26.3	2.71	<0.01

*p*-significance.

**Table 4:** Testing the influence of selected factors on changes in locomotion activity of dams during estrus compared to the reference period before and after estrus.

Factor of Influence	DF	Mean Squares	F Value	*p*	R^2^
Herd	1	61,411.22	1.66	0.1978	0.38
Parity	1	494,926.75	13.41	0.0003	
Lactation stage	3	54,804.75	1.48	0.2181	
Milk yield	1	43,330.67	1.13	0.2792	
Factor of individual	207	41,663.69	1.17	0.1522	

*p*—significance; R^2^—reliability of the estimate; DF—degrees of freedom.

**Table 5:** Testing the influence of selected factors on changes in the locomotion activity of cows during estrus compared to the reference period before and after estrus using the Post Hoc Test.

Indicator	Level	Mean	Number	Duncan Grouping
Herd	1	297.37	340	A
	2	277.61	293	A
Parity	heifers	392.90	78	AB
	primiparous	324.00	237	A
	multiparous	260.42	318	B
Lactation stage	heifers	2921.90	78	A
	up to 80 days	299.72	236	A
	81 to 150 days	262.42	180	A
	over 151 days	299.50	139	A
Milk yield	<34.29 kg of milk	289.31	356	A
	≥34.29 kg of milk	286.83	277	A

Significance level α = 0.05.

## Data Availability

The data presented in this study are available on reasonable request from the corresponding author.
